# Acceptance and Use of Generative AI in Clinical Rehabilitation: An Interdisciplinary Survey of US Rehabilitation Professionals

**DOI:** 10.1016/j.arrct.2025.100571

**Published:** 2025-12-10

**Authors:** Malachy J. Clancy, Erin R. Pletcher, Alison Bell, Travis Pollen, Robert Dekerlegand

**Affiliations:** a Department of Occupational Therapy, Thomas Jefferson University, Philadelphia, PA; b Department of Exercise Science, Thomas Jefferson University, Philadelphia, PA; c Department of Physical Therapy, Thomas Jefferson University, Philadelphia, PA

**Keywords:** Athletic trainers, Generative artificial intelligence, Occupational therapy, Physical therapists, Rehabilitation, Speech language pathology

## Abstract

**Objective:**

To examine rehabilitation professionals’ current clinical use and practice patterns of generative artificial intelligence (GenAI) and examine differences between users and nonusers.

**Design:**

Cross-sectional mixed-methods.

**Setting:**

Online survey.

**Participants:**

A total of 408 professionals responded, with 264 complete surveys (64.7% completion) analyzed quantitatively and 311 valid open-ended responses analyzed qualitatively. The participants included occupational therapists, physical therapists, speech language pathologists, clinical exercise physiologists, and athletic trainers in the United States.

**Interventions:**

Not applicable.

**Main Outcome Measures:**

Researcher-designed survey based on the Technology Acceptance Model and Unified Theory of Acceptance and Use of Technology.

**Results:**

Responses were received from all regions of the US, and were primarily occupational therapists (n=136, 51.5%) and women (n=214, 81.1%). Most participants identified as nonusers (n=154, 58.3%), whereas 41.6% (n=101) reported using GenAI some or most of the time. ChatGPT was the most common platform identified by all participants (n=241, 91.3%). GenAI was recognized by rehabilitation professionals as a tool to support patient care and administrative tasks. Users were significantly more likely than nonusers to perceive GenAI as useful in 5 of 7 domains (*P*<.001) and easy to use in 3 of 6 domains (*P*<.001). Logistic regression demonstrated that peer influence was the strongest predictor of use (*P*<.001, odds ratio 6.16, 95% CI, 2.94-12.93).

**Conclusions:**

Perceptions of usefulness, ease of use, and the social influence of peers significantly influence rehabilitation professionals’ adoption and the use of GenAI within clinical practice.

## Highlights

• The influence of peers using generative artificial intelligence (GenAI) was associated with a 6-fold adoption of GenAI in practice.
• Users reported using GenAI to primarily support administrative tasks and not direct patient care.
• Nonusers described potential use for documentation, but reported far more negative concerns.

Generative artificial intelligence (GenAI) represents a subset of AI capable of creating novel content, including text, images, audio, and code, through sophisticated machine learning models.^1^ Given the widespread availability of robust products such as ChatGPT and DALL-E, the interest in GenAI has resulted in a sharp rise in public usage,^2^^,^^3^ and there is a consensus that GenAI will transform health care.^4^ Recent work has explored how health care workers envision the use of GenAI for administrative support, clinical decision assistance, resource use optimization, and greater access to information.^5^, ^6^, ^7^, ^8^, ^9^ Despite growing awareness of GenAI capabilities among health care professionals, there is a gap between its perceived potential value and clinical adoption.^5^^,^^10^ This disconnect may be influenced by multiple factors and perceived threats that impede the translation of GenAI to clinical practice.

Although many health care professionals are aware of GenAI and recognize its potential benefits, the actual application in clinical practice remains infrequent.^5^^,^^10^ Understanding rehabilitation professionals’ current perspectives and usage regarding GenAI is crucial for developing appropriate guidelines and training to successfully integrate this technology in the clinic. Current studies exploring rehabilitation professionals’ GenAI perceptions have focused on AI broadly rather than the specific capabilities of GenAI,^7^^,^^10^^,^^11^ examined a single discipline rather than the multidisciplinary practitioners characteristic of rehabilitation practice,^5^^,^^7^^,^^12^ and investigated isolated GenAI platforms rather than the diverse technologies available.^5^

An understanding of rehabilitation professionals’ perceptions and actual use of GenAI across available technologies, professional disciplines, and practice settings can facilitate its responsible adoption in practice. Therefore, the purpose of this study was to (1) examine current GenAI usage patterns and applications among rehabilitation professionals and (2) identify factors differentiating GenAI users from nonusers.

## Methods

### Study design and participants

This mixed-methods, cross-sectional online survey sampled rehabilitation professionals within the United States. The survey was distributed to athletic trainers (ATs), clinical exercise physiologists, occupational therapists (OTs, including assistants), physical therapists (PTs, including assistants), and speech language pathologists (SLPs) between April 8, 2025, and May 28, 2025, using convenience and purposive sampling. Potential participants were invited to complete the survey by e-mail invitations sent through professional networks and rosters of clinical educators within the targeted academic disciplines. In addition, recruitment flyers were distributed at professional conferences, and online announcements were posted on relevant national online professional community groups and social media networks. Snowball sampling was used by encouraging recipients to forward the study recruitment announcement within their professional networks. Follow-up e-mails and social media postings were redistributed 2 weeks later. The study was approved by the Institutional Review Board of the parent university (iRISID-2024-0914)*.*

Participants provided consent at the start of the survey. Credentialed and/or licensed professionals from 1 of the 5 targeted professions were eligible. As such, all participants were 18 years of age or older at the time of survey completion. No other specific exclusion criteria were applied.

### Survey development

The survey was developed by the study authors with experience in both survey development and GenAI, and was based on previous work.^13^ The survey was guided by the Technology Acceptance Model (TAM)^14^ and the Unified Theory of Acceptance and Use of Technology (UTAUT).^15^ The TAM posits that use is influenced by its perceived ease of use and usefulness. The UTAUT extends this to include social, environmental, and personal factors.^16^ Participants answered questions specifically about using GenAI in their “clinical work,” defined as activities specific to “patient care, education, research, administration, and advocacy” to focus responses to the survey.

Before distribution, the survey was reviewed by 3 external faculty with academic expertise in the use of GenAI and technology to maximize validity, and was tested for functionality by a small group of student assistants. Branching logic was incorporated into the electronic survey with adjusted question language for participants who use or do not use GenAI in their clinical practice. The final survey used a combination of Likert scale and open-ended questions to assess participants’ demographics, perceived usefulness, perceived ease of use, and social influences of GenAI. The final electronic survey (supplemental appendix S1, available online only at http://www.archives-pmr.org/) was distributed via QualtricsXM^a^ (SAP).

### Data analysis

Data from all submitted surveys were exported from QualtricsXM to SPSS version 29.0.2^b^ and subsequently reviewed for completion and eligibility. After review, participants with at least 75% response completion were included in the qualitative analysis, and only those with 100% response completion were included for quantitative analyses. Nominal and ordinal data were summarized by frequency counts and relative percentages; continuous data were summarized by mean (SD) or median (range). Likert scale responses were combined into 2 categories (disagree/strongly disagree or agree/strongly agree) for ease of interpretation, as overall fewer respondents selected “strongly agree” or “strongly disagree.” Data were first analyzed descriptively, given the exploratory nature of this study. Differences in response distributions between users and nonusers were compared with chi-square tests for nominal and ordinal data with significance set at α=0.05.

Potential covariates associated with the logit (user vs nonuser) were identified with bivariate statistics and were retained for binary logistic modeling if statistically significant (*P*<.05). Sex, age, and years of experience were also included in statistical modeling, informed by the UTAUT.^15^ The assumption of linearity of each of 2 continuous predictor variables (age and years of experience) with the logit was assessed using the Box-Tidwell procedure with Bonferroni correction. Separate multivariate logistic regression models were generated for each of the survey components (TAM usefulness, TAM ease of use, and facilitators) combined with age, years of experience, and sex. A final model was subsequently developed, combining all covariates significantly associated with the logit. All regression modeling used the forced-entry method with the significance set at α=0.05.

Summative qualitative content analysis was used to analyze open-ended responses. Content analysis is a qualitative method for categorizing textual data to identify patterns and derive meaning.^17^ Two study authors (A.B. and T.P.) independently reviewed the free-text data and collaboratively developed an initial coding framework. Responses were then coded independently by both researchers, achieving 69.2% inter-rater agreement. Through discussion, the codebook (supplemental appendix S2, available online only at http://www.archives-pmr.org/) was refined, and differences were resolved through discussion until consensus was reached.

## Results

### Participants

A total of 408 participants responded to the survey, with 328 open-ended responses (of which 311 were valid for qualitative analysis). In the final analysis, 264 full responses (64.7% completion rate) were included for quantitative analysis, with responses received from all representative disciplines. Table 1 illustrates the demographic data for the participants included in the quantitative analysis (supplemental table S1 [available online only at http://www.archives-pmr.org/] provides full demographic data for qualitative analysis). The mean age was 40.7±10.6y, with 15.3±10.2y of professional experience. Women made up 81.1% (n=214) of respondents. Primary clinical disciplines were OTs (n=136, 51.5%), PTs (n=79, 29.9%), ATs (n=27, 10.2%), SLPs (n=15, 5.7%), clinical exercise physiologists (n=2, 0.8%), and “other” (n=2, 1.9%). Within each discipline, most respondents worked in direct patient care (n=207, 88.3%), followed by administration (n=23, 8.7%), academic (n=20, 7.6%), research (n=3, 1.1%), and “other” (n=11, 4.2%).

**Table 1:** Participant demographics and use of generative artificial intelligence in clinical practice (N=264).

	All N=264	Nonusers n=154 (58.3%)	Users n=110 (41.7%)
Use of AI, n (%)			
All the time	9 (3.4%)	–	9 (8.2%)
Most of the time	12 (4.5%)	–	12 (10.9%)
Some of the time	89 (33.7%)	–	89 (80.9%)
None of the time	154 (58.3%)	154 (100%)	–
Age mean (SD)	40.7 (10.6)	41.05 (10.8)	40.24 (10.3)
Years of clinical exp mean (SD)	15.3 (10.2)	16.0 (10.4)	14.5 (10.1)
Primary discipline, n (%)			
Athletic training	27 (10.2%)	19 (12.3%)	8 (7.3%)
Occupational therapy	136 (51.5%)	78 (50.6%)	58 (52.7%)
Physical therapy	79 (29.9%)	47 (30.5%)	32 (29.1%)
Speech language pathology	15 (5.7%)	5 (3.2%)	10 (9.1%)
Clinical exercise physiology	2 (0.8%)	2 (1.3%)	0 (0%)
Other	5 (1.9%)	3 (1.9%)	2 (1.8%)
Work status, n (%)			
Full-time	233 (88.3%)	135 (87%)	98 (89.1%)
Part-time	14 (5.3%)	8 (5.2%)	6 (5.5%)
Perdiem	17 (6.4%)	11 (7.1%)	6 (5.5%)
Work role, n (%)			
Direct patient care	207 (88.3%)	135 (87.7%)	72 (65.5%)
Researcher	3 (1.1%)	2 (1.3%)	1 (0.9%)
Administration	23 (8.7%)	10 (6.5%)	13 (11.8%)
Academic	20 (7.6%)	6 (3.9%)	14 (12.7%)
Other	11 (4.2%)	1 (0.6%)	10 (9.1%)
US region of practice, n (%)			
Midwest	20 (7.6%)	10 (6.5%)	10 (9.1%)
Northeast	189 (71.6%)	118 (76.6%)	71 (64.5%)
South	28 (10.6%)	14 (9.1%)	14 (12.7%)
West	19 (7.2%)	8 (5.2%)	11 (1.0%)
Missing	8 (3.0%)	4 (2.6%)	4 (3.6%)
Practice setting, n (%)			
Acute care hospital	55 (20.8%)	39 (25.3%)	16 (14.5%)
Acute rehabilitation	24 (9.1%)	18 (11.7%)	6 (5.5%)
Athletic/sports team	11 (4.2%)	6 (3.9%)	5 (4.5%)
Community-based practice	12 (4.5%)	2 (1.3%)	10 (9.1%)
Early intervention	7 (2.7%)	4 (2.6%)	3 (2.7%)
Home health	2 (0.8%)	2 (1.3%)	0 (0%)
Industry	1 (0.4%)	1 (0.6%)	0 (0%)
Other	52 (19.7%)	26 (16.9%)	26 (23.6%)
Outpatient	41 (15.5%)	27 (17.5%)	14 (12.7%)
Schools	54 (20.5%)	26 (16.9%)	28 (25.5%)
Subacute rehabilitation	5 (1.9%)	3 (1.9%)	2 (1.8%)
Highest level of education, n (%)			
Associates	1 (0.4%)	1 (0.6%)	0 (0.0%)
Bachelors	32 (12.1%)	17 (11.0%)	15 (13.6%)
Masters	126 (47.7%)	84 (54.5%)	42 (38.2%)
Clinical doctorate	90 (34.1%)	44 (28.6%)	46 (41.8%)
Academic doctorate	15 (5.7%)	8 (5.2%)	7 (6.4%)
Professional organization member, n (%)			
Yes	188 (71.2%)	103 (66.9%)	85 (77.3%)
No	76 (28.8%)	51 (33.1%)	25 (22.7%)
Sex, n (%)			
Male	50 (18.9%)	35 (22.7%)	15 (13.6%)
Female	214 (81.1%)	119 (77.3%)	95 (86.4%)
Race and ethnicity, n (%)			
Arabic/Middle Eastern	2 (0.8%)	1 (0.6%)	1 (0.9%)
Asian	13 (4.9%)	7 (4.5%)	6 (5.5%)
Black or African American	10 (3.8%)	6 (3.9%)	4 (3.6%)
Hispanic/Latino/Spanish	4 (1.5%)	2 (1.3%)	2 (1.8%)
Other	1 (0.4%)	1 (0.6%)	0 (0%)
Prefer not to say	4 (1.5%)	3 (1.9%)	1 (0.9%)
White	236 (89.4%)	138 (89.6%)	98 (89.1%)

NOTE. This table presents only participants included in the quantitative analysis.

### Usage patterns and applications

Over half (58.3%, n=154) of the respondents indicated using GenAI “none of the time” in clinical practice, 33.7% (n=89) reported “some of the time,” and 7.9% (n=21) “most of” or “all of the time.” The prevalence of using GenAI varied by discipline, with 52.7% (n=58) of OTs, 29.1% (n=32) of PTs, 9.1% (n=10) of SLPs, 7.3% (n=8) of ATs, and 1.8% (n=2) of “others” identifying as users. Of the users of GenAI, the most frequent platforms were ChatGPT (n=92, 83.6%), Google/Gemini (n=41, 37.3%), and Microsoft Copilot (n=21, 19.1%). Respondents who do not use GenAI had also heard of ChatGPT (n=149, 96.8%), Google/Gemini (n=112, 72.7%), and Microsoft Copilot (n=46, 29.9%).

**Fig 1:**
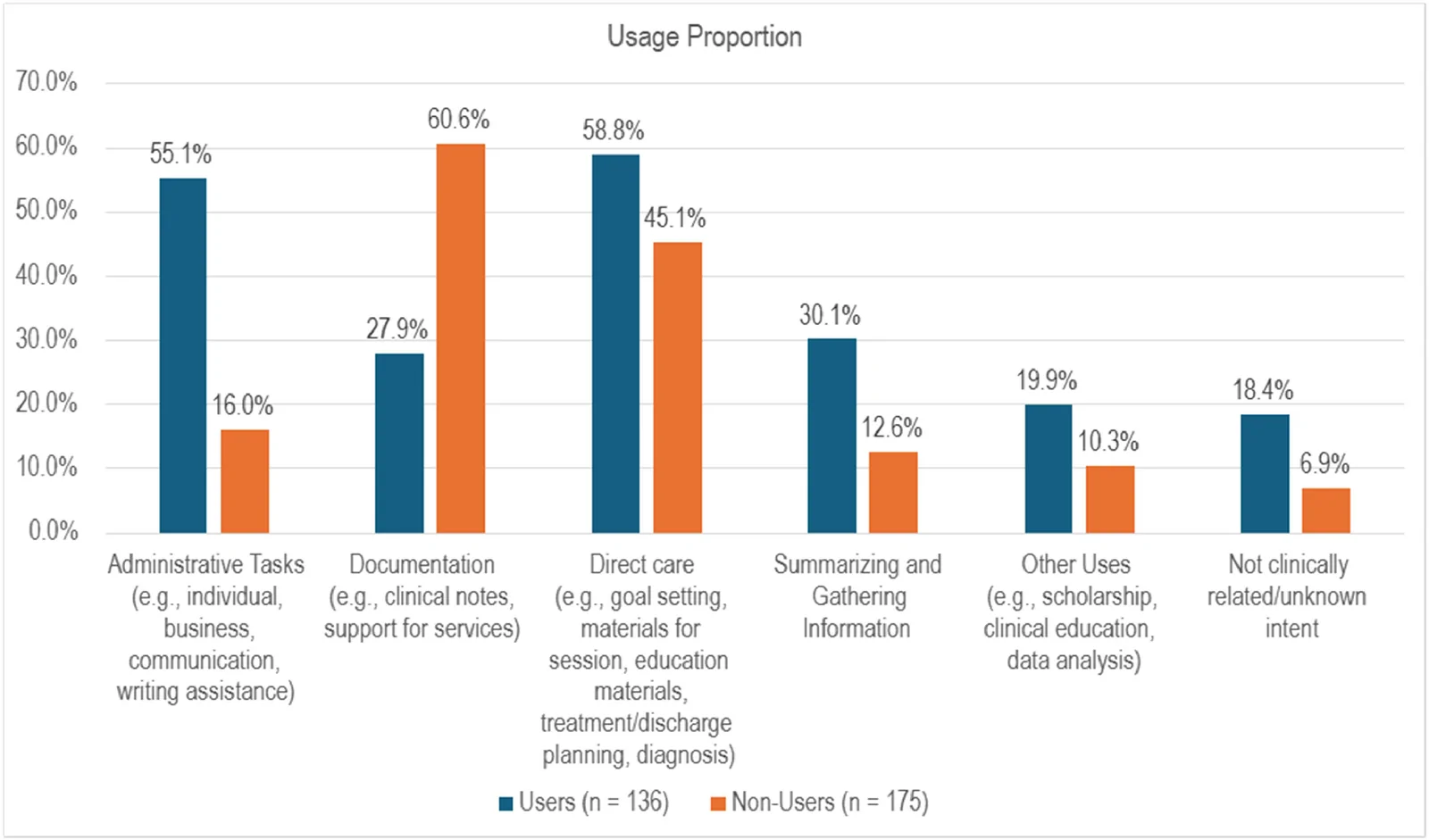
Proportion of reported GenAI usage between users and nonusers (n=311).

Through free-text responses, 99.2% of users (n=136) and 91.6% of nonusers (n=175) described how they currently use or could use GenAI in the clinical environment. Respondents frequently identified more than one use of GenAI, resulting in frequency counts that are larger than the sample size. The most commonly reported use of GenAI in the clinical setting was to support patient care tasks (n=80, 58.8%), such as treatment planning (n=37, 27.2%) and patient and care partner education (n=15, 11.0%). Users also reported that GenAI supported the administrative aspects of practice (n=75, 55.1%), such as communicating with the interprofessional team and the patient (n=30, 22.1%), and general support for writing (n=27, 19.9%). Nonusers of GenAI described potential use for documentation (n=106, 60.6%) more frequently than users (n=38, 27.9%) and identified the potential for GenAI to provide support for patient care (n=79, 45.1%), including treatment planning (n=45, 25.7%) and patient and caregiver education (n=16, 9.16%) (fig 1). Nonusers of GenAI reported far more negative comments or concerns about GenAI use (n=27, 15.4%) than users (n=4, 2.9%) (table 2).

**Table 2:** Free-text responses regarding GenAI.

Free-Text Response
“I don’t believe it would be useful at all and actually a detriment for critical thinking.” Nonuser 1 Response
“I don't think it would assist my clinical work; if only it would add to the burden of making patients use it instead of talking to them.” Nonuser 2 Response
“At this time, I don’t see how generative AI would reduce my work burden; to me, it seems like just another thing that would be added to what I already have to do.” Nonuser 3 Response
“Generative AI HIPAA Compliant app (NABLA) has been rolled out at our health system for creating transcriptions and AI-generated clinical notes - but I have not found it helpful as of yet for reducing time demands.” User 1 Response

### Perceptions of GenAI usefulness and ease of use

Most survey participants reported that GenAI is both useful (table 3) and easy to use (table 4). Significant differences were seen in the distribution of responses in 6 of 7 questions related to GenAI perceived usefulness and 4 of 6 attributed to its perceived ease of use. Overall, users perceived GenAI as more useful and easier to use as compared with nonusers. A weak association was noted between GenAI usage and its perceived ability to accomplish clinical tasks (Cramer’s V=0.173, *P*=.005), whereas moderate associations were noted with perceptions on the impact on clinical performance, clinical effectiveness, making work easier, ability to learn, and overall usefulness (Cramer’s V range 0.238-0.286, *P*<.001). GenAI usage was weakly associated with clinicians’ perceptions on ease of getting GenAI to do the desired intention (Cramer’s V=0.173, *P*=.005) and moderately associated with perceptions related to understandable interactions, flexibility, and overall ease of use (Cramer’s V range 0.204-0.274, *P*<.001).

**Table 3:** Perceived usefulness of generative artificial intelligence in clinical practice.

Question Domain*	All N=264	Nonusers n=154	Users n=110	χ^2^ *P* Value	Cramer’s V
Accomplishes clinical tasks (%)					
Disagree/strongly disagree	24.2	30.5	15.5	.005	0.173
Agree/strongly agree	75.8	69.5	84.5		
Improves clinical performance (%)					
Disagree/strongly disagree	36.7	48.1	20.9	<.001	0.278
Agree/strongly agree	63.3	51.9	79.1		
Increases clinical productivity (%)					
Disagree/strongly disagree	32.6	34.4	30.0	.450	0.046
Agree/strongly agree	67.4	65.6	70.0		
Enhances clinical effectiveness (%)					
Disagree/strongly disagree	39.8	51.3	23.6	<.001	0.279
Agree/strongly agree	60.2	48.7	76.4		
Makes clinical work easier (%)					
Disagree/strongly disagree	27.3	37.7	12.7	<.001	0.276
Agree/strongly agree	72.7	62.3	87.3		
Is useful in clinical (%)					
Disagree/strongly disagree	19.3	27.3	8.2	<.001	0.238
Agree/strongly agree	80.7	72.7	91.8		
Helps my ability to learn clinically (%)					
Disagree/strongly disagree	38.3	50.0	21.8	<.001	0.286
Agree/strongly agree	61.7	50.0	78.2		

⁎ The general question domain is provided in the table. See supplemental appendix S1 for the full question stem, as verbiage was adapted for users versus nonusers.

**Table 4:** Perceived ease of use of generative artificial intelligence in clinical practice.

Question Domain*	All N=264	Nonusers n=154	Users n=110	χ^2^ *P* Value	Cramer’s V
Learning to use is easy in clinic (%)					
Disagree/strongly disagree	24.2	27.9	19.1	.099	0.102
Agree/strongly agree	75.8	72.1	80.9		
Easy to get it to do intention in clinic (%)					
Disagree/strongly disagree	44.7	51.9	34.5	.005	0.173
Agree/strongly agree	55.3	48.1	65.5		
Interaction is understandable in clinic (%)					
Disagree/strongly disagree	40.2	50.6	25.5	<.001	0.253
Agree/strongly agree	59.8	49.4	74.5		
Flexible to interact with in clinic (%)					
Disagree/strongly disagree	37.5	48.7	21.8	<.001	0.274
Agree/strongly agree	62.5	51.3	78.2		
Easy to become skillful in GenAI in clinic (%)					
Disagree/strongly disagree	31.4	32.5	30.0	.670	0.026
Agree/strongly agree	68.6	67.5	70.0		
Easy to use in clinic (%)					
Disagree/strongly disagree	32.2	40.3	20.9	<.001	0.204
Agree/strongly agree	67.8	59.7	79.1		

^-^The general question domain is provided in the table. See supplemental appendix S1 for the full question stem as verbiage was adapted for users versus nonusers.

Responses related to survey questions about facilitators of using GenAI and perceived accuracy are shown in table 5. Compared with nonusers, significantly more users reported they had the required resources, worked for supportive supervisors/organizations, and their respected peers used GenAI. Moderate to relatively strong associations were noted between GenAI use and peer influence (Cramer’s V=0.463, *P*<.001), available resources (Cramer’s V=0.394, *P*<.001), and organizational support (Cramer’s V=0.315, *P*<.001). Nonusers more frequently perceived GenAI as a threat to their job as compared with users (24% vs 10%, *P*=.004), which was weakly associated with usage (Cramer’s V=0.179, *P*=.004). Users reported that they can recognize if/when GenAI provides inaccurate information most of the time (58.2%) or all of the time (25.5%).

**Table 5:** Influential factors of generative artificial intelligence usage in clinical practice.

Question Domain*	All N=264	Nonusers n=154	Users n=110	χ^2^ *P* Value	Cramer’s V
Peers I respect use GenAI (%)					
Disagree/strongly disagree	58.0	77.3	30.9	<.001	0.463
Agree/strongly agree	42.0	22.7	69.1		
Supervisors support use of GenAI (%)					
Disagree/strongly disagree	11.4	13.6	8.2	.003	0.212
Unsure	54.9	61.0	46.4		
Agree/strongly agree	33.7	25.3	45.5		
Organization supports use of GenAI (%)					
Disagree/strongly disagree	10.6	14.3	5.5	<.001	0.315
Unsure	55.7	64.3	43.6		
Agree/strongly agree	33.7	21.4	50.9		
I have the resources I need to use GenAI (%)					
Disagree/strongly disagree	61.7	77.9	39.1	<.001	0.394
Agree/strongly agree	38.3	22.1	60.9		
GenAI will threaten my job in the future (%)					
Disagree/strongly disagree	81.8	76.0	90.0	.004	0.179
Agree/strongly agree	18.2	24.0	10.0		
GenAI provides accurate information (%)					
None of the time	4.2	6.5	0.9	.121	0.149
Some of the time	54.5	55.2	53.6		
Most of the time	40.2	37.0	44.5		
All of the time	1.1	1.3	0.9		
I can recognize if/when GenAI provides inaccurate information (%)					
None of the time	1.1	1.3	0.9	<.001	0.272
Some of the time	29.9	40.3	15.5		
Most of the time	49.2	42.9	58.2		
All of the time	19.7	15.6	25.5		
I am responsible for the accuracy of GenAI (%)					
Share responsibility/I am not/Unsure	12.1	14.9	8.2	.097	0.102
Fully responsible	87.9	85.1	91.8		
I know how to indicate the use of GenAI (%)					
No	61.4	64.9	56.4	.159	0.087
Yes	38.6	35.1	43.6		
I indicate when I use GenAI (%)					
Most/Some/None of the time	57.6	37.7	85.5	<.001	0.477
All of the time	42.4	62.3	14.5		

⁎ The general question domain is provided in the table. See supplemental appendix S1 for the full question stem as verbiage was adapted for users versus nonusers.

### Factors associated with GenAI use

Table 6 presents the statistically significant results of the final binary logistic regression analysis determining the influence of demographic, attitudinal, and organizational characteristics on predicting usage. The model was statistically significant, χ^2^(17)=117.69, *P*<.001; it explained 48.4% (Nagelkerke R^2^) of the variance and correctly classified 79.2% of cases. Of the 17 predictor variables, 6 were statistically significant: age, years of experience, sex, and responses to these 3 statements: “Peers I respect are using GenAI in their clinical work,” “The organization where I work is supportive of GenAI in my clinical work,” and “I have the resources I need to use GenAI in my clinical work.” Respected peers’ usage (odds ratio 6.16; 95% CI, 2.94-12.93) was the strongest predictor of GenAI use in the clinic. A receiver operating characteristic curve was used to assess how well the model distinguishes between nonusers and users. The area under the curve was 0.861 (95% CI, 0.816-0.907), indicating an excellent level of discrimination.^18^ Stratified analyses for the TAM usefulness, TAM ease of use, and facilitators are presented in supplemental tables S2-S4 (available online only at http://www.archives-pmr.org/), and for the full model in supplemental table S5 (available online only at http://www.archives-pmr.org/).

**Table 6:** Binary logistic regression on predicting users of generative artificial intelligence.

			95% CI	
Variable	Sig.	Odds Ratio	Lower	Upper
Age	0.035	1.095	1.006	1.192
Clinical experience (y)	0.019	0.901	0.826	0.983
Sex	0.023	2.695	1.149	6.318
Peers I respect are using generative AI in their clinical work	<0.001	6.166	2.940	12.932
The organization where I work is supportive of the use of generative AI in my clinical work	0.045	3.354	1.028	10.945
I have the resources I need to use generative AI in my clinical work	0.002	3.201	1.551	6.609

NOTE: The table displays the statistically significant variables that were identified in the final multivariable logistic model developed by combining all covariates that were associated with the logit. The units for age and clinical experience are in 1-year increments.

## Discussion

This cross-sectional survey study examined the perceptions, beliefs, and usage patterns of GenAI among rehabilitation professionals across multiple rehabilitation disciplines through a mixed-methods approach. The results demonstrate significant differences in perceived ease of use, perceived usefulness, and presence of facilitators between users of GenAI and nonusers in clinical practice. We found that less than half (41.6%) of rehabilitation professionals are using GenAI in clinical practice as a peripheral supplementary tool within their clinical work. Both users and nonusers recognize GenAI’s potential for improving efficiency, even if they differ in other perceptions related to its clinical value. Users appear to view GenAI not just as a task-completion tool, but as a learning resource that enhances their clinical knowledge and skills. This study is the first to our knowledge to explore these perceptions across multiple rehabilitation disciplines within the United States.

The most common actual use of GenAI was to complete administrative tasks. Over half of users and nearly half of nonusers identified that GenAI could be used as an assistive tool for creating tools and resources for direct care, such as supporting treatment planning. The clinical usage of GenAI reported by the respondents in this study is consistent with the reported or envisioned use of GenAI identified in recent literature.^5^, ^6^, ^7^, ^8^, ^9^ Previous work supports the perceived clinical usefulness of GenAI in reducing administrative burden and improving efficiencies, such as scheduling appointments,^19^ documenting and updating medical records,^7^ and synthesizing information.^20^ Many clinicians also see GenAI as an assistive tool that can augment, rather than replace, human expertise. Earlier studies have shown the use of GenAI to improve diagnostic accuracy^21^^,^^22^ and monitor patient progress and provide feedback.^11^

Consistent with the predictions of the TAM,^14^ the perceived ease of use and perceived usefulness were greater for users of GenAI, suggesting that direct experience with GenAI may positively influence perceptions of this technology. In the domain of usefulness, the only question that did not demonstrate statistical significance was “increases clinical productivity.” Rehabilitation professionals often report both ethical and practical challenges in managing productivity standards.^23^^,^^24^ These standards are based on the number of billable units, face-to-face time spent with a patient, or the number of visits achieved,^25^ which are unlikely to change with the use of GenAI. It is possible that respondents interpreted productivity in this way, rather than overall time spent in the work environment. The only area in the perceived ease of use domain that did not demonstrate a statistically significant difference between users and nonusers was “easy to become skillful in GenAI,” with both groups perceiving GenAI as easy to use. The consensus that both groups found it easy to become skillful in GenAI, despite a lack of resources, may reflect the relatively low barrier to entry when using GenAI.

Consistent with the UTAUT, the influence of the social and organizational environment was a key factor associated with usage. We found that users were 6 times more likely to adopt GenAI if a peer they respect is using the technology, and 3 times more likely if they have the needed resources or organizational support for GenAI use in their clinical environment. This suggests that peer adoption and professional modeling significantly influence individual decisions to use GenAI, highlighting the importance of social learning and professional networks in technology adoption.

Both users and nonusers generally disagreed with the idea that GenAI will threaten their job future. This perception is consistent with other surveys of health care workers who did not report believing current technology has the capacity to do their clinical work,^5^ in part because of the interpersonal process being a hallmark of patient-centered care.^26^ Nevertheless, some respondents still reported concerns over the technology, and previous studies in health care professionals have described a fear of job displacement or the loss of valued clinical tasks.^6^^,^^8^ It remains unclear if this perceived “substitution crisis” may be a barrier to the widespread adoption and implementation of the technology in clinical practice.^6^

Although GenAI is easily accessible for general use, a major challenge is the lack of advanced understanding and knowledge regarding AI technologies needed for skilled clinical application.^10^^,^^27^ Similar to this study, in other studies many rehabilitation professionals reported not having the resources to use GenAI in their clinical work and indicated they did not have the appropriate training or were not prepared to implement GenAI in practice.^6^^,^^27^ This knowledge gap is particularly notable in specific applications like rehabilitation, where PTs’ knowledge of AI usage in rehabilitation was found to be lower than their general AI knowledge.^11^^,^^12^ Other studies have proposed training curricula to address GenAI literacy, including enabling users to understand and interact with GenAI models, with recommendations that rehabilitation science curricula begin to teach these skills.^7^^,^^28^

### Study limitations

We employed both purposive and snowball sampling, and although our sample is large, it includes an over-representation of respondents from the Northeastern United States and from the OT profession. Sampling and response bias are possible, as the discourse of GenAI may have influenced the desire for participation within this study. In addition, it should be noted that our sample included nonphysician professionals as the sampling pools were derived from the professional rehabilitation networks of the authors. As such, the results are specific to our sample. The design of our study limits our ability to establish causality, such as whether prior training may lead to increased use of GenAI, which we did not explore. The rapidly changing evolution of GenAI, including updated models such as GPT-5, may increase the future use of this technology in clinical practice. Although the categorical fixed response of survey items in our questionnaire allowed us to characterize usage patterns and compare groups, such questions may have oversimplified professional attitudes toward the actual use or nonuse of the technology. However, our qualitative data present additional depth and context to support our findings. We performed regression modeling to explore factors associated with use; however, we were unable to account for unobserved confounding variables, such as organizational policies or institutional culture that may influence GenAI adoption.

## Conclusions

The results of this national survey highlight that perceptions of usefulness, ease of use, and the social influence of peers significantly influence professionals’ GenAI usage patterns within clinical practice. Both opportunities and challenges are associated with the adoption and use of this emerging technology in rehabilitation practice, and the results underscore the need for future training to influence adoption behaviors. We encourage future work to explore the acceptance and use of GenAI with a larger sample of more diverse practitioners. In addition, exploring the impact of training, institutional policies, and the ability to access GenAI in health care institutions may influence the adoption and use of GenAI in clinical practice.

## Suppliers

a. QualtricsXM; SAP.
b. SPSS, version 29.0; IBM.

## Disclosure

The investigators have no financial or nonfinancial disclosures to make in relation to this project.

## Appendix Supplementary materials

Image, application 1

Image, application 2

Image, application 3

## Footnotes

Supplementary material associated with this article can be found, in the online version, at doi:10.1016/j.arrct.2025.100571.
