# Supplementary material for: Acceptance and Use of Generative AI in Clinical Rehabilitation: An Interdisciplinary Survey of US Rehabilitation Professionals
Source: Arch Rehabil Res Clin Transl. 2025 Dec 10;8(3):100571. doi: 10.1016/j.arrct.2025.100571 (PMC13591089; doi:10.1016/j.arrct.2025.100571)
Supplement: Supplementary file 1 [file mmc1.docx]

**Supplemental Appendix S1:** Survey used to assess perceptions and use of generative artificial intelligence in clinical work.

When answering the following questions, ***"clinical work"*** includes patient care, education, research, administration, and advocacy.

1. How frequently do you use generative AI in your clinical work?

All of the time	Most of the time	Some of the time	None of the time

**Part 1**

The following questions seek to understand your perceptions of the ***usefulness / potential usefulness*** of generative AI in your clinical work.

2. Using generative AI ***helps/would help*** me accomplish my clinical work more quickly.

Strongly Agree	Agree	Disagree	Strongly Disagree

3. Using generative AI ***improves/would improve*** my clinical work performance.

Strongly Agree	Agree	Disagree	Strongly Disagree

4. Using generative AI in my clinical work ***increases/would increas*e** my productivity.

Strongly Agree	Agree	Disagree	Strongly Disagree

5. Using generative AI in my clinical work ***enhances/would enhance*** my effectiveness.

Strongly Agree	Agree	Disagree	Strongly Disagree

6. Using generative AI ***makes/would make*** my clinical work easier.

Strongly Agree	Agree	Disagree	Strongly Disagree

7. Using generative AI ***is useful/would be useful*** in my clinical work.

Strongly Agree	Agree	Disagree	Strongly Disagree

8. Using generative AI in my clinical work ***helps/would help*** my ability to learn.

Strongly Agree	Agree	Disagree	Strongly Disagree

9. List examples of how you ***have used generative AI/think generative AI could be used*** in your clinical work.

**Part 2**

The following questions seek to understand your perceptions on the ***ease of use/potential ease of use*** of generative AI in your clinical work.

10. Learning to use generative AI in my clinical work ***is/would be*** easy for me.

Strongly Agree	Agree	Disagree	Strongly Disagree

11. I ***find/would find*** it easy to get generative AI to do what I want it to do in my clinical work.

Strongly Agree	Agree	Disagree	Strongly Disagree

12. My interaction with generative AI ***is/would be*** clear and understandable in my clinical work.

Strongly Agree	Agree	Disagree	Strongly Disagree

13. I ***find/would find*** generative AI flexible to interact with in my clinical work.

Strongly Agree	Agree	Disagree	Strongly Disagree

14. It ***is/would be*** easy for me to become skillful at using generative AI in my clinical work.

Strongly Agree	Agree	Disagree	Strongly Disagree

15. I ***find/would find*** generative AI easy to use in my clinical work.

Strongly Agree	Agree	Disagree	Strongly Disagree

**Part 3**

When answering the questions in this section, consider the ***use / potential use*** of generative AI in your clinical work.

16. Peers I respect are using generative AI for their clinical work.

Strongly Agree	Agree	Disagree	Strongly Disagree

17. Supervisors in my organization ***are/would be*** supportive of the use of generative AI in my clinical work. 

Strongly Agree	Agree	Unsure	Disagree	Strongly Disagree

18. The organization where I work ***is/would be*** supportive of the use of generative AI in my clinical work.

Strongly Agree	Agree	Unsure	Disagree	Strongly Disagree

19. I have the resources I ***need/would need*** to use generative AI in my clinical work.

Strongly Agree	Agree	Disagree	Strongly Disagree

20. Advancement in generative AI will threaten my job in the future.

Strongly Agree	Agree	Disagree	Strongly Disagree

21. ***When I use/If I were to use*** generative AI in my clinical work, it ***provides/would provide*** accurate information.

All of the time	Most of the time	Some of the time	None of the time

22. ***When I use/If I were to use*** generative AI in my clinical work, I ***can/would be able to*** recognize if/when it provides inaccurate information.

All of the time	Most of the time	Some of the time	None of the time

23. ***When using/If I were to use*** generative AI in my clinical work, I ***am/would be*** responsible for the accuracy of the information it provides.

I am fully responsible

I share responsibility with the AI system

I am not responsible

Not sure

24. I ***know/would know*** how to indicate the use of generative AI in my clinical work.

Yes

No

25. ***I indicate when I use generative AI in my clinical work / If I were to use generative AI in my clinical work, I would indicate when I use it.***

All of the time	Most of the time	Some of the time	None of the time

26. ***Which AI products are you using in your clinical work / Which of the following AI products have you heard of?*** (Select all that apply)

- ChatGPT
- ChatPDF
- Google/Gemini
- Claude
- Microsoft Copilot/Bing Chat
- Meta Llama
- Perplexity
- Adobe Firefly
- Midjourney
- I have not heard of any listed
- Other:

**Part 4**

27. I work primarily as a/an:

- Athletic Trainer (ATC)
- Certified Occupational Therapist Assistant (COTA)
- Clinical Exercise Physiologist (CEP)
- Occupational Therapist (OTR)
- Physical Therapist (PT)
- Physical Therapist Assistant (PTA)
- Speech Language Pathologist (SLP)
- Speech Language Pathologist Assistant (SLPA)
- Other:

28. In my clinical role, I currently work:

- Full-Time
- Part-Time
- Per-Diem
- Retired
- Seeking employment

29. My primary role at work is: 

- Direct Patient Care
- Academic
- Researcher
- Administration
- Other:

30. How many years of clinical experience do you have?

- Years of experience:
- Prefer not to answer

31. What state do you practice in? 

DROP DOWN: Alabama ... Wyoming

32. What setting/area do you primarily practice in?

- Acute Care Hospital
- Acute Rehabilitation Facility
- Athletics / Sports Team
- Community Based Practice
- Early Intervention
- Fitness Center
- Home Health
- Industry
- Military
- Outpatient Clinic (Physician Practice)
- Schools
- Sub-Acute Rehabilitation
- Other:

33. What is your highest level of education?

- Associates Degree (AS, AAS, etc)
- Bachelors Degree (BS, BSHS, etc.)
- Master's Degree (MOT, MPT, MS, MBA, etc.)
- Clinical Doctorate (DAT, OTD, DPT, SLPD, DrOT, DHSc, etc.)
- Academic Doctorate (PhD, ScD, EdD)

34. Are you a member of your profession's national organization?

- Yes
- No

35. How old are you?

- Age:
- Prefer not to answer

36. What is your gender identity?

- Male
- Female
- Non-binary / third gender
- Prefer not to say

37. What is your race and ethnicity? (Select all that apply)

- White
- Black or African American
- American Indian or Alaska Native
- Asian
- Native Hawaiian or Pacific Islander
- Hispanic, Latino, or Spanish
- Arabic or Middle Eastern
- Prefer not to specify
- Other:
