# Supplementary material for: Acceptance and Use of Generative AI in Clinical Rehabilitation: An Interdisciplinary Survey of US Rehabilitation Professionals
Source: Arch Rehabil Res Clin Transl. 2025 Dec 10;8(3):100571. doi: 10.1016/j.arrct.2025.100571 (PMC13591089; doi:10.1016/j.arrct.2025.100571)
Supplement: Supplementary file 2 [file mmc2.docx]

**SUPPLEMENTAL INFORMATION**

**Supplemental Table S1:** Full demographics and use of generative artificial intelligence in clinical practice

|  | **All** | **Non-Users** | **Users** |
| --- | --- | --- | --- |
| **Use of AI** n (%)  All the time  Most of the time  Some of the time  None of the time | N=328  10 (3.0)  15 (4.6)  112 (34.1)  191 (58.2) | n=191  -  -  -  191 (100.0) | n=137  10 (7.3)  15 (11.0)  112 (81.2)  - |
| **Age** mean (SD) | n=265  40.7 (10.6) | n=155  41.0 (10.8) | n=110  40.2 (10.3) |
| **Years of Clinical Exp** mean (SD) | n=265  15.4 (10.3) | n=155  16.0 (10.4) | n=110  14.5 (10.1) |
| **Primary Discipline** n (%)  Athletic Training  Occupational Therapy  Physical Therapist  Speech-Language Pathology  Clinical Exercise Physiology  Other | n=299  28 (9.4)  152 (50.8)  93 (31.1)  17 (5.7)  3 (1.0)  6 (2.0) | n=176  20 (11.4)  86 (48.9)  56 (31.8)  7 (4.0)  3 (1.7)  4 (2.3) | n=123  8 (6.5)  66 (53.7)  37 (30.1)  10 (8.1)  0 (0.0)  2 (1.6) |
| **Work Status** n (%)  Full-Time  Part-Time  Per-Diem | n=299  265 (88.6)  17 (5.7)  17 (5.7) | n=176  155 (88.1)  10 (5.7)  11 (6.3) | n=123  110 (89.4)  7 (5.7)  6 (4.9) |
| **Work Role** n (%)  Direct Patient Care  Researcher  Administration  Academic  Other | n=299  233 (77.9)  3 (1.0)  25 (8.4)  24 (8.0)  14 (4.7) | n=176  155 (88.1)  2 (11.4)  11 (6.3)  6 (3.4)  2(1.1) | n=123  78 (63.4)  1 (0.8)  14 (11.4)  18 (14.6)  12 (9.8) |
| **U.S. Region of Practice** n (%)  Midwest  Northeast  Pacific  South  West | n=328  20 (6.1)  215 (65.5)  12 (3.7)  29 (8.8)  23 (8.1) | n= 191  10 (5.2)  134 (70.2)  8 (4.2)  14 (7.3)  3 (1.6) | n=137  10 (7.3)  81 (59.1)  4 (2.9)  15 (10.9)  8 (5.8) |
| **Practice Setting** n (%)  Acute Care Hospital | 59 (19.7) | 42 (23.9) | 17 (13.8) |
| Acute Rehabilitation | 27 (9.0) | 20 (11.4) | 7 (5.7) |
| Athletics/Sports Team | 12 (4.0) | 7 (4.0) | 5 (4.1) |
| Community-Based Practice | 15 (5.0) | 2 (1.1) | 13 (10.6) |
| Early Intervention | 8 (2.7) | 5 (2.8) | 3 (2.4) |
| Home Health | 2 (0.7) | 2 (1.1) | 0 (0) |
| Industry | 1 (0.3) | 1 (0.1) | 0 (0) |
| Other | 57 (19.1) | 29 (16.5) | 28 (22.8) |
| Outpatient | 46 (15.4) | 30 (17.0) | 16 (13.0) |
| Schools | 64 (21.4) | 32 (18.2) | 32 (26.0) |
| Sub-Acute Rehabilitation | 8 (2.7) | 6 (3.4) | 2 (1.6) |
|  |  |  |  |
| **Highest Level of Education** n (%)  Associates  Bachelors  Masters  Clinical Doctorate  Academic Doctorate | n=299  1 (0.3)  38 (12.7)  139 (46.5)  104 (34.8)  17 (5.7) | n=176  1 (0.1)  23 (13.1)  92 (52.3)  52 (29.5)  8 (4.5) | n=123  0 (0.0)  15 (12.2)  47 (38.2)  52 (42.3)  9 (7.3) |
| **Professional Organization Member** n (%)  Yes  No | n=299  210 (70.2)  89 (29.8) | n=176  117 (66.5)  59 (33.5) | n=123  93 (75.6)  30 (24.4) |
| **Gender Identity** n (%)  Man  Woman  Non-binary/third gender  Prefer not to say | n=299  52 (17.4)  240 (80.3)  1 (0.3)  6 (2.0) | n=176  37 (21.0)  135 (76.7)  1 (0.5)  3 (1.7) | n=123  15 (12.2)  105 (85.4)  0 (0.0)  3 (2.4) |
| **Race and Ethnicity** n (%) | n=299 | n=176 | n=123 |
| American Indian/Alaskan Native | 2 (0.7) | 1 (0.6) | 1 (0.8) |
| Arabic/Middle Eastern | 5 (1.7) | 2 (1.1) | 3 (2.4) |
| Asian | 15 (5.0) | 9 (5.1) | 6 (4.9) |
| Black or African American | 12 (4.0) | 7 (4.0) | 5 (4.1) |
| Hispanic/Latino/Spanish | 4 (1.3) | 2 (1.1) | 2 (1.6) |
| Native Hawaiian/Pacific Islander | 1 (0.3) | 1 (0.6) | 0 (0) |
| Other | 1 (0.3) | 1 (0.6) | 0 (0) |
| Prefer not to say | 14 (4.7) | 8 (4.5) | 6 (4.9) |
| White | 258 (86.3) | 153 (86.9) | 105 (85.4) |
|  |  |  |  |

**Note**: Data within the table is presented for participants in both the qualitative and quantitative analysis

**Supplemental Table S2**: Binary Logistic Regression for TAM Perceived Usefulness in Predicting Users

|  |  |  |  |  |  |  | **95% CI** | |
| --- | --- | --- | --- | --- | --- | --- | --- | --- |
| **Variable** | **B** | **S.E.** | **Wald** | **df** | **Sig.** | **OR** | **Lower** | **Upper** |
| Age | 0.044 | 0.037 | 1.461 | 1 | 0.227 | 1.045 | 0.973 | 1.123 |
| Gender | 0.807 | 0.365 | 4.897 | 1 | 0.027 | 2.242 | 1.097 | 4.582 |
| Years of experience | -0.058 | 0.038 | 2.405 | 1 | 0.121 | 0.943 | 0.876 | 1.016 |
| Using generative AI helps me accomplish my clinical work more quickly. | -0.193 | 0.446 | 0.187 | 1 | 0.666 | 0.825 | 0.344 | 1.977 |
| Using generative AI improves my clinical work performance. | 0.507 | 0.386 | 1.724 | 1 | 0.189 | 1.661 | 0.779 | 3.542 |
| Using generative AI in my clinical work enhances my effectiveness | 0.361 | 0.368 | 0.960 | 1 | 0.327 | 1.434 | 0.697 | 2.950 |
| Using generative AI makes my clinical work easier. | 0.699 | 0.478 | 2.140 | 1 | 0.143 | 2.013 | 0.789 | 5.137 |
| Using generative AI is useful in my clinical work | 0.035 | 0.578 | 0.004 | 1 | 0.952 | 1.036 | 0.333 | 3.218 |
| Using generative AI in my clinical work helps my ability to learn. | 0.706 | 0.356 | 3.936 | 1 | 0.047 | 2.025 | 1.009 | 4.067 |
| Constant | -3.347 | 1.109 | 9.110 | 1 | 0.003 | 0.035 |  |  |

**Note**: The units for age and clinical experience are in one-year increments. (OR = Odds Ratio)

**Supplemental Table S3**: Binary Logistic Regression for the TAM Ease of Use on Predicting Users

|  |  |  |  |  |  |  | **95% CI** | |
| --- | --- | --- | --- | --- | --- | --- | --- | --- |
| **Variable** | **B** | **S.E.** | **Wald** | **df** | **Sig.** | **OR** | **Lower** | **Upper** |
| Age | 0.061 | 0.036 | 2.774 | 1 | 0.096 | 1.062 | 0.989 | 1.141 |
| Gender | 0.858 | 0.360 | 5.679 | 1 | 0.017 | 2.357 | 1.164 | 4.772 |
| Years of clinical experience | -0.069 | 0.037 | 3.475 | 1 | 0.062 | 0.933 | 0.867 | 1.004 |
| I find it easy to get generative AI to do what I want it to do in my clinical work | -0.079 | 0.391 | 0.041 | 1 | 0.840 | 0.924 | 0.429 | 1.988 |
| My interaction with generative AI is clear and understandable in my clinical work | 0.622 | 0.415 | 2.240 | 1 | 0.134 | 1.862 | 0.825 | 4.204 |
| I find generative AI flexible to interact with in my clinical work | 0.792 | 0.388 | 4.168 | 1 | 0.041 | 2.209 | 1.032 | 4.727 |
| I find generative AI easy to use in my clinical work. | 0.197 | 0.424 | .216 | 1 | 0.642 | 1.218 | 0.530 | 2.796 |
| Constant | -3.439 | 1.123 | 9.379 | 1 | 0.002 | 0.032 |  |  |

**Note**: The units for age and clinical experience are in one-year increments. (OR = Odds Ratio)

**Supplemental Table S4**: Binary Logistic Regression for the Facilitators on Predicting Users

|  |  |  |  |  |  |  | **95% CI** | |
| --- | --- | --- | --- | --- | --- | --- | --- | --- |
| **Variable** | **B** | **S.E.** | **Wald** | **df** | **Sig** | **OR** | **Lower** | **Upper** |
| Age | 0.089 | 0.039 | 5.248 | 1 | 0.022 | 1.093 | 1.013 | 1.180 |
| Gender | 0.821 | 0.405 | 4.112 | 1 | 0.043 | 2.272 | 1.028 | 5.021 |
| Years of clinical experience | -0.103 | 0.040 | 6.609 | 1 | 0.010 | 0.902 | 0.833 | 0.976 |
| Peers I respect are using generative AI in their clinical work | 1.786 | 0.343 | 27.151 | 1 | <.001 | 5.967 | 3.048 | 11.683 |
| Supervisors in my organization are supportive of the use of generative AI in my clinical work | -0.766 | 0.548 | 1.954 | 1 | 0.162 | 0.465 | 0.159 | 1.361 |
| The organization where I work is supportive of the use of generative AI in my clinical work. | 0.878 | 0.547 | 2.572 | 1 | 0.109 | 2.405 | 0.823 | 7.029 |
| I have the resources I need to use generative AI in my clinical work | 1.433 | 0.336 | 18.227 | 1 | <0.001 | 4.190 | 2.171 | 8.090 |
| Advancement in generative AI will threaten my job in the future. | -1.472 | 0.461 | 10.179 | 1 | 0.001 | 0.230 | 0.093 | 0.576 |
| Constant | -4.267 | 1.189 | 12.877 | 1 | <.001 | .014 |  |  |

**Note**: The units for age and clinical experience are in one-year increments. (OR = Odds Ratio)

**Supplemental Table S5**: Full Model Output for Binary Logistic Regression on Predicting Users

|  |  |  |  |  |  |  | **95% CI** | |
| --- | --- | --- | --- | --- | --- | --- | --- | --- |
| **Variable** | **B** | **S.E.** | **Wald** | **df** | **Sig.** | **OR** | **Lower** | **Upper** |
| Age | 0.091 | 0.043 | 4.423 | 1 | 0.035 | 1.095 | 1.006 | 1.192 |
| Years of clinical experience | -0.104 | 0.045 | 5.502 | 1 | 0.019 | 0.901 | 0.826 | 0.983 |
| Gender | 0.991 | 0.435 | 5.199 | 1 | 0.023 | 2.695 | 1.149 | 6.318 |
| Using generative AI helps me accomplish my clinical work more quickly. | -0.389 | 0.546 | 0.507 | 1 | 0.476 | 0.678 | 0.232 | 1.977 |
| Using generative AI improves my clinical work performance. | 0.899 | 0.464 | 3.760 | 1 | 0.052 | 2.457 | 0.990 | 6.095 |
| Using generative AI in my clinical work enhances my effectiveness | 0.101 | 0.449 | 0.051 | 1 | 0.821 | 1.107 | 0.459 | 2.668 |
| Using generative AI makes my clinical work easier. | 0.350 | 0.562 | 0.389 | 1 | 0.533 | 1.420 | 0.472 | 4.269 |
| Using generative AI is useful in my clinical work | -0.096 | 0.664 | 0.021 | 1 | 0.885 | 0.908 | 0.247 | 3.334 |
| Using generative AI in my clinical work helps my ability to learn. | 0.825 | 0.434 | 3.607 | 1 | 0.058 | 2.281 | 0.974 | 5.344 |
| I find it easy to get generative AI to do what I want it to do in my clinical work | 0.111 | 0.488 | 0.052 | 1 | 0.820 | 1.117 | 0.429 | 2.908 |
| My interaction with generative AI is clear and understandable in my clinical work | 0.533 | 0.535 | 0.996 | 1 | 0.318 | 1.705 | 0.598 | 4.861 |
| I find generative AI flexible to interact with in my clinical work | 0.035 | 0.519 | 0.005 | 1 | 0.946 | 1.036 | 0.375 | 2.864 |
| I find generative AI easy to use in my clinical work. | -0.112 | 0.527 | 0.045 | 1 | 0.831 | 0.894 | 0.318 | 2.512 |
| Peers I respect are using generative AI in their clinical work | 1.819 | 0.378 | 23.173 | 1 | <0.001 | 6.166 | 2.940 | 12.932 |
| Supervisors in my organization are supportive of the use of generative AI in my clinical work | -1.198 | 0.616 | 3.783 | 1 | 0.052 | 0.302 | 0.090 | 1.009 |
| The organization where I work is supportive of the use of generative AI in my clinical work. | 1.210 | 0.603 | 4.023 | 1 | 0.045 | 3.354 | 1.028 | 10.945 |
| I have the resources I need to use generative AI in my clinical work | 1.164 | 0.370 | 9.896 | 1 | 0.002 | 3.201 | 1.551 | 6.609 |
| Constant | -5.955 | 1.390 | 18.350 | 1 | <0.001 | 0.003 |  |  |

**Note**: The units for age and clinical experience are in one-year increments. (OR = Odds Ratio)
