# Supplementary material for: Acceptance and Use of Generative AI in Clinical Rehabilitation: An Interdisciplinary Survey of US Rehabilitation Professionals
Source: Arch Rehabil Res Clin Transl. 2025 Dec 10;8(3):100571. doi: 10.1016/j.arrct.2025.100571 (PMC13591089; doi:10.1016/j.arrct.2025.100571)
Supplement: Supplementary file 3 [file mmc3.docx]

**Supplemental Appendix S2**: Codebook

| **Category** | **Subcategory** | **Description** | **Specific Rules** |  |
| --- | --- | --- | --- | --- |
| Administrative Tasks | Individual administrative tasks | Tasks completed by **individual** clinicians that support but are not part of direct patient care. | Includes scheduling, meeting minutes, | |
|  | Business practice management | Tasks that support the **business operations**, such as marketing, budget, business plan, quarterly reports, social media (marketing), recruitment, policy, and procedures. Staff management including scheduling and performance reviews | Includes marketing (including social media), budgets, business plans, policy, and procedures. Includes staff management, such as scheduling and performance reviews | |
|  | Communication | Emails and other forms of communication with the interprofessional team, patients, and families. |  | |
|  | Writing assistance | General organization, writing, grammar, outlines or proofreading without a defined purpose | No defined purpose for writing task | |
| Documentation | Documentation of services | Direct documentation of client activities |  | |
|  | Support for services | Insurance and reimbursement-related documentation | Includes LMN, insurance reviews, appeals and prior authorization | |
|  |  |  |  |  |
| Patient care | Goal setting | Establishing treatment objectives and outcomes |  | |
|  | Materials for the session | Development of materials or worksheets used in the session |  | |
|  | Patient/caregiver education materials | Education resources | Includes the materials given to patients for HEP. | |
|  | Treatment planning | Development of intervention strategies and plans | Includes the idea development for HEP | |
|  | Discharge planning | Transition or aftercare planning |  | |
|  | Diagnosis | Support to develop clinical diagnosis |  | |
| No Category | Information gathering | Using to find information, answer questions, or research topics | Includes AI generated summaries | |
|  | Scholarship | Development of materials for publication, presentation. Includes applications for external funding |  | |
|  |  |  |  |  |
|  |  |  |  |  |
|  |  |  |  |  |
|  | Clinical education | Materials to support mentorship and education in the clinical environment |  | |
|  | Data analysis | Assistance with quantitative or qualitative data analysis tasks |  | |
|  | Uncategorized | Not related to clinical role or Unknown intent |  | |
|  | Other | Clinical use of GenAI is not defined by codebook | Includes Job hunting, studying for a certification exam | |
|  | Negative/Concern | Response expressing concern, limitations, or negative experience |  | |
